# Whole-exome sequencing detected a novel *AIFM1* variant in a Han-Chinese family with Cowchock syndrome

**DOI:** 10.1186/s41065-023-00282-z

**Published:** 2023-05-12

**Authors:** Chenyu Wang, Zhaojing Lin, ZhuangZhuang Yuan, Tieyu Tang, Liangliang Fan, Yihui Liu, Xuan Wu

**Affiliations:** 1 Department of Neurology, Affiliated Hospital of Yangzhou University, Yangzhou, 225001 China; 2 Department of Cell Biology, The School of Life Sciences, Central South University, Changsha, 410013 China; 3 Department of anesthesia, The Second of Xiangya Hospital of Central South University, Changsha, 410078 China

**Keywords:** AIFM1, Charcot-Marie-Tooth disease, Molecular diagnosis, CMTX4

## Abstract

Charcot-Marie-Tooth disease(CMT) is a hereditary peripheral neuropathy, characterized by progressive distal hypoesthesia and amyotrophia. CMT is characterized by an X- linked recessive inheritance pattern. The apoptosis-inducing factor mitochondria associated-1 (AIFM1) is the main pathogenic gene of the X-linked recessive Charcot-Marie-Tooth disease-4 with or without cerebellar ataxia (CMTX4), also known as Cowchock syndrome. In this study, we enrolled a family with CMTX from the southeast region of China and identified a novel AIFM1 variant (NM_004208.3: c.931C>G; p.L311V) using whole exon sequencing technology. The results of our study may also be useful for genetic counseling, embryo screening of in vitro fertilization embryos, and prenatal genetic diagnosis.

## Introduction

Cowchock syndrome, also known as Charcot-Marie-Tooth disease linked 4 (CMTX4) is a rare peripheral sensory-motor neuropathy characterized by an X-linked recessive inheritance pattern. Cowchock syndrome develops slowly in the neonatal period to a severe onset in early childhood showing distal muscle weakness and atrophy of the peroneal muscle group, sensorineural hear loss and cognitive impairment.

The *AIFM1* gene has been associated with X-linked chromosome recessive inherited disease that presents with a spectrum of auditory and sensory neuropathy phenotypes [1, 2]. The *AIFM1* gene encodes a 67 kDa mitochondrial flavin adenine dinucleotide (FAD)-dependent oxidoreductase, which plays a role in oxidative phosphorylation and redox control in healthy cells. The FAD-dependent nicotinamide adenine dinucleotide (NADH) oxidase consists of six domains, a mitochondrial-specific targeting sequence of 54 amino acids at its N-terminal, inner membrane sorting signal domain, two FAD domains, an NADH binding domain, and a C-terminal domain (Fig. 2C).

In this study, we reported a family with X-linked *AIFM1*-associated disease and detected a novel missense variant (NM_004208.3: c.931C>G: p.L311V) in *AIFM1*. Affected individuals presented severe symptoms, including axonal sensory-motor neuropathy damage, distal muscle weakness, and atrophy, exhibited classic symptoms of Charcot-Marie-Tooth (CMT) disease, such as deafness and intellectual disability, which are consistent with the typical symptoms of the Cowchock syndrome, as described in previous studies. Thus, these findings demonstrated that missense variant in AIFM1 may lead to the syndrome.

## Material and methods

### Study participants and clinical evaluation

Members of a four-generation Han Chinese pedigree with CMTX4, consisting of 14 individuals, were enrolled for genetic screening at the Department of Neurology, Affiliated Hospital of Yangzhou University (Fig. 2A). The peripheral venous blood of three available family members was sampled for the genetic study. All medical records of healthcare, routine physical, and pure tone audiometry report were collected. All participating subjects obtained informed consent. The study was approved by the Institutional Review Board of Affiliated hospital of Yangzhou University.

### DNA extraction, exome sequencing, quality control and filtering

Preparation of genomic DNA from peripheral venous blood of patients referenced from a previously published article. Whole-exome sequencing (WES) and Quality control were from Berry Genomics (Beijing, China). Filtering method referenced form article [3]. Suspicious variants were scored according to the American College of Medical Genetics (ACMG). The potential causative variants of CMT were screened.

### Mutation Analysis

The bioinformatics program Mutation Taster (http://www.mutationtaster.org) was used to predict the effects of mutations detected by WES. The mutation most likely to lead to the disease was verified by Sanger sequencing. Methods referenced from published article [3]. We model the protein structure by Swiss-model (https://swissmodel.expasy.org/interactive), and construct amino acid point from leucine311 to valine311 by pymol. Meanwhile, we measure the changes of distance between molecules, intermolecular angle and surface charge after variant.

## Case report

**Fig. 1:**
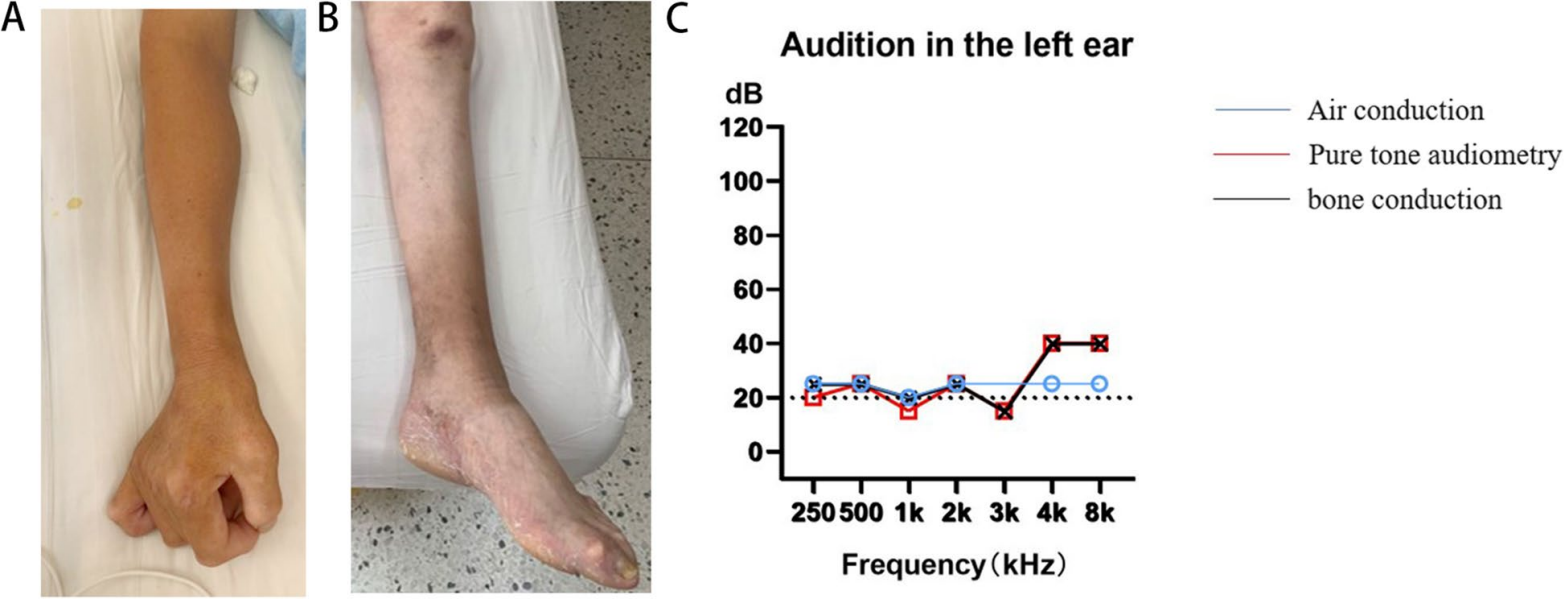
**A** The bilateral forearm muscles and the muscles of both hands were significantly atrophied. **B** The bilateral lower limb muscles were significantly atrophied, especially the calves with high arched feet. **C** Pure tone audiometry report shows that hearing impaired of proband

We report a 42-year-old male proband who experienced slowly progressive myasthenia and muscle atrophy, characterized by prone to falling while walking, progressive weakness of both lower extremities and muscle atrophy at the age of 10 years. Thinning of the calf, contracture of the ankle joint. The clinical presentation was consistent with CMT. The proband was born after a normal pregnancy to the mother with no apparent abnormalities during labor or the neonatal period. His motor milestones were unremarkable until the age of 10, after which his parents began to notice that he fell frequently, his calves became thinner, he struggled to walk for long periods of time, with progressive aggravation, and later he had difficulty climbing stairs and squatting; The proband showed muscular atrophy worsens after the age of 20 and has to be wheelchair-bound since the age of 30. In the past 5 years, the fine movement of the hands was poor, the hand muscles were atrophied, and it was difficult to hold objects (Fig. 1A). During the first few years, his parents considered his intellectual development to be normal, but gradually found that his writing and numeracy skills began to decline, he showed learning difficulties at school, dropped out of school at home, and developed hearing loss after the age of 15. Physical examination revealed patient with a thin body, wheelchair limitations, and scoliosis deformity. Neurological examinations showed marked reductions in memory, calculation, and executive function, with a Montreal Cognitive Assessment MoCA score of 16. Speech is not fluent, bilateral pupils are equal and round, sensitive to light reflex, facial lines are symmetrical, central tongue extension, no tongue muscle atrophy. Hearing of proband lost on the coarse side (Fig. 1C). The bilateral scapular muscles of proband were mildly atrophied, the bilateral forearm muscles and the extremity muscles of proband were significantly atrophied, especially the calves with high arched feet (Fig. 1B). The proximal muscle strength of both upper extremities is grade 4, the distal muscle strength is grade 3, and the muscle strength of both lower extremities is grade 2. The muscle tone of the limbs is low, the tendon reflexes disappear, the depth and superficial sensation disappears, the bilateral pathological signs and meningeal irritation signs are negative. Neurophysiological examination of the proband showed axonal polyneuropathy of the upper (median and ulnar nerves) and lower limbs (common peroneal and tibial nerves), the lower limb motor nerve conduction complicated motor action potential did not elicit, and the amplitude of the upper limb was significantly reduced (Table 1). Sensory nerve action potentials disappeared. The main findings of the investigation of the nerve conduction were consistent with the characteristics of length dependence in severe axonal polyneuropathy (Table 2). Electromyography demonstrated denervation potentials and reduced motor unit potentials in the affected muscles. Charcot-Maria-Tooth Examination Score is 24. We then investigated the family history of the proband (IV:3), which indicated that his brother (IV:4) shows more severe symptoms than his brother’s.

**Table 1:** Nerve Conduction studies summary of the proband

Side	Motor/Sensor	Nerve	Latency(ms)	Amplitude(mv)	Conduction xelocity(m/s)
Right	Motor	Median Wrist--APB	10.0	0.91	NP
		Ulnar Wrist--ADM	NP	NP	NP
		Peroneus Ankle--EDC	NP	NP	NP
		Tibial Ankle--AH	NP	NP	NP
		Musculocutaneous Erb-BB	6.35	1.21	NP
		Femoral inguinal -VM	NP	NP	NP
	Sensor	Median Finger3-Wrist	NP	NP	NP
		Ulnar Finger5-Wrist	NP	NP	NP
		superficial peroneal Calf-Ankle	NP	NP	NP
		Sural Calf-Heel	NP	NP	NP

*APB* Abductor pollicis brevis, *ADM* Abductor digiti minimi, *BB* Biceps brachii, *EDC* Extensor digitorum brevis, *AH* Abductor halluces, *VM* Vastus medialis

**Table 2:** Needle EMG result of the proband

Side	Muscle	Insertional activity (Normal/Increased,N/I)	Spntaneous (Normal/Increased,N/I)			MUAP (Normal/Increased,N/I)				Recruitment (Reduced/NP)
Right/Left (R/L)			Fib	Psw	Fas	Dur	Amp	Poly		
L	FDI	NP	NP	NP	NP	NP	NP	NP	NP	NP
	BB	I	I	I	NP	I	I	I		R
	MA	N	I	I	NP	I	I	I		R
	TRA	I	NP	I	NP	I	I	I		R
R	ILP	I	NP	I	NP	I	I	I		R
	VL	I	NP	I	NP	I	I	I		R
	GM	NP	NP	NP	NP	NP	NP	NP	NP	NP
	AT	NP	NP	NP	NP	NP	NP	NP	NP	NP

*Fib* Fibrillation, *PSW* Pasitive sharo wave, *Dur* Duration, *Amp* Amplitude, *Poly* Polyphasics, *FDI* First dorsal interosseous, *BB* Biceps brachii, *MA* Masseter, *TRA* Trapezius, *ILP* Lliopsoas, *VL* VM, Vastus medialis, *GM* Gastrocenemius, *AT* Tibialis anterior

**Fig. 2:**
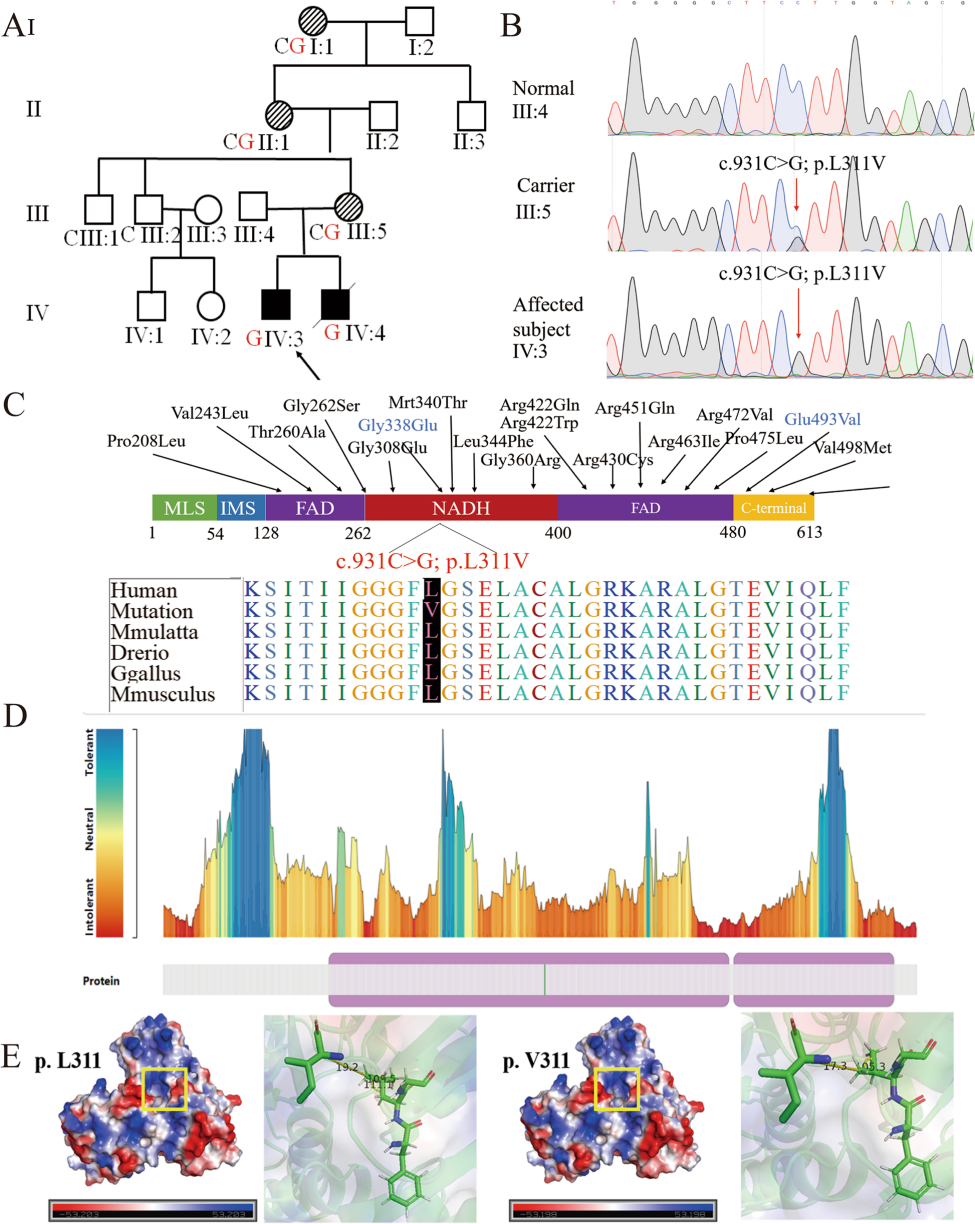
**A** Pedigree of the patient with CMTX4. Family members are identified by generation and number. Squares indicate male family members; circles indicate female member; symbol with bias indicate carrier; arrow indicate proband. C means wild-type sequence, G means c.931C>G in patient, CG means carrier. Arrow indicate the proband. **B** Sanger Sequencing confirmed the variation in patients; Sequence analysis of new mutations of c.931C>G; p.L311V. (Black=G, Red=T, Blue=C, Green=A) **C** The domain of AIFM1, at the mutation position of c.931C>G; p.L311V, Conservative analysis and Comparison of the functional domains: Compared sequence of amino acid, mutation sites (red domain) show a high degree of conservatism in different species. Known AIFM1 mutation with CMTX4 marked by red. **D** Meta Dome predicted the variant amino site is intolerant. **E** Surface charge diagram of AIFM1; Diagrams displaying the position of Leu311 and Val 311, respectively. yellow dashed lines show Distance between adjacent molecules

Considering of genetic factor, fourteen individuals of a four-generation Han-Chinese pedigree with CMTX4 were enrolled for genetic screening at the Department of Neurology, Affiliated Hospital of Yangzhou University (Fig. 2A). Peripheral venous blood of three family members was sampled for genetic analysis. All medical records of healthcare, routine physical, and pure tone audiometry reports were collected. Informed consent was obtained from all the participants. All participating subjects obtained informed consent. The study was approved by the Institutional Review Board of the Affiliated Hospital of the Yangzhou University.

We performed whole exon sequencing on the proband and parents and screened for novel missense variants (NM_004208: c.931C>G: p.L311V) in *AIFM1*, which was validated by Sanger sequencing in the proband (Fig. 2B). In previous studies, *AIFM1* has been confirmed to be closely related to the occurrence of CMTX4. Sanger sequencing confirmed that the mother of the proband (III-5) also carried the variant (NM_004208: c.931C>G: p.L311V). Polyphen25, and MutationTaster6 predicted that the variant is pathogenic and conforms to the law of co-segregation (Fig. 2C). The variant is c.931C>G, and the protein change occurs at position 311, which is a intolerant site predicted by Meta dome, converting leucine to valine acid (Fig. 2D). The novel variant meetings the following criteria from the ACMG guidelines: PM1, PM2, PM5, PP1, PP3. Genetic testing can be useful for clinical diagnosis of many subtypes of diseases represented by CMT. Next generation sequencing to identify disease subtypes and further improve the quality of clinical methods. The finding of variant expands the spectrum of variant in AIFM1. In prenatal diagnosis, this finding improves the accuracy of clinical genetics.

## Discussion

In this study, we used whole exon sequencing to explore the causative gene for a family with Cowchock syndrome and identified a novel missense variant (c.931C>G:p.L311V) in *AIFM1*. This variant was confirmed by Sanger sequencing. Affected family members (IV-3 and IV-4) not only exhibited classic CMT symptoms, but also presented deafness and intellectual disability, which are consistent with the typical symptoms of the Cowchock syndrome. The variant is located in the NADH binding domain (263–400), and the protein change occurs at position 311, converting leucine to valine acid. The amino acid hydrogen bond angle changes from 109° to 105°, after variant; The distance to adjacent molecules is shortened, which affects a slight change in charge (Fig. 2E).

The human *AIFM1* gene encodes a 67 kDa mitochondrial FAD-dependent oxidoreductase that plays a role in oxidative phosphorylation and redox control [4, 5]. Previous studies have shown that AIFM1 interacts with the oxidoreductase mitochondrial intermembrane space import and assembly protein 40 (MIA40) in cultured cells and mouse tissues. Eighty percent of *AIFM1* defects lead to the loss of Mia40 and coenzyme I assembly errors, which leads to muscle atrophy, astrogliosis, and progressive neurodegeneration in a mouse model [2]. Our study is consistent with previous studies showing that variants in *AIFM1* lead to Cowchock syndrome.

The FAD-dependent oxidoreductase consists of six domains, including a mitochondrial-specific targeting sequence of 54 amino acids at its N-terminal, inner membrane sorting signal domain (55–128), two FAD domains (129–262, 401–480), an NADH binding domain (263–400), and a C-terminal domain (481–608) [6, 7]. The enzyme functions as a NADH oxidoreductase and regulators of apoptosis. In response to apoptotic stimuli, AIFM1 is released from the mitochondrial membrane space into the cytoplasm and nucleus. In this process, it binds to DNA in a sequence-independent manner. AIFM1 plays a critical role in caspase-independent pyknotic cell death in hydrogen peroxide-exposed cells and is also related to electronic transfer activities.

Susin (1999) identified and cloned the apoptosis-inducing factor *AIF*, which induces cell nucleus apoptosis *in vitro*. Susin cloned a mouse homolog of *AIF*, which has 92% amino acid identity with human proteins [7, 8]. Both mouse and human full-length proteins contain two mitochondrial localization sequences and two putative nuclear localization signals. *AIF* is usually confined in the inner mitochondrial membrane and transfers to the nucleus when inducing apoptosis. Ghezzi (2010) determined that AIFM1 binds to FAD and is attached to the inner mitochondrial membrane through the N-terminal transmembrane domain, acting as NADH oxidase [6, 9].

Presently, 19 variants of the *AIFM1* gene have been found, most of which occur in two FAD-binding and NADH-binding domains. In our case, the amino acid change caused by the variant c.931C>G:p.L311V) was present in the NADH-binding domain and the protein change occurred at position 311, converting leucine to valine. This conversion caused protein charge changes and may disturb AIFM1 as the NADH oxidase attached to the inner mitochondrial membrane.

In conclusion, we enrolled a Han-Chinese family with CMT. Whole exome sequencing and Sanger sequencing detected a missense variant (NM_004208.3:c.931C>G:p.L311V) of AIFM1 in the CMT patients and absent in the healthy members. Our discovery may assist in genetic counseling, embryonic screening of *in vitro* fertilized embryos, and prenatal genetic diagnosis. And this result contributes to potential gene-targeted therapies.

## Data Availability

All supporting data of this article are included in the submitted manuscript.

## Abbreviations

CMT: Charcot-Marie-Tooth
CMTX4: Charcot-Marie-Tooth disease linked 4
AIFM1: apoptosis-inducing factor mitochondria associated-1
CMTX: X-linked recessive inheritance pattern
NADH: nicotinamide adenine dinucleotide
